# Single-Molecule Chemistry Part II: Pathway Analysis of the Oxidation of Guanine to 8-Oxo-7,8-dihydroguanosine in an Oligonucleotide Hybrid

**DOI:** 10.3390/molecules31101564

**Published:** 2026-05-08

**Authors:** Jens Sobek, Marco Schmidt, Stephan Landgraf, Aaron Fleming, Kiyohiko Kawai, Ralph Schlapbach

**Affiliations:** 1 Functional Genomics Center Zurich, University and ETH Zurich, 8057 Zurich, Switzerland; marco.schmidt@fgcz.ethz.ch (M.S.); ralph.schlapbach@fgcz.ethz.ch (R.S.); 2 Institute of Physical and Theoretical Chemistry, Graz University of Technology, 8010 Graz, Austria; landgraf@tugraz.at; 3 Department of Chemistry, University of Utah, Salt Lake City, UT 84112-0850, USA; afleming@chem.utah.edu; 4 Department of Life Science and Technology, School of Life Science and Technology, Institute of Science Tokyo, Yokohama 226-8501, Japan; kawai.k@life.isct.ac.jp

**Keywords:** single-molecule measurement, G oxidation, oligonucleotides, Cy3, pathway analysis, hybridisation, zero-mode waveguides, fluorescence, SPR, intermediates

## Abstract

In a short 11 nt dye-labelled oligonucleotide hybrid, we investigated the oxidation of a single guanine, G, to 8-oxo-7,8-dihydroguanosine, 8oG, a reaction proceeding via two reaction pathways. In single-molecule fluorescence traces, we were able to detect the conversion of G to 8oG and assign different reaction pathways. Reactions are initiated by a photoinduced electron transfer to the dye, creating a G radical cation, G^•+^, which is in equilibrium with the neutral G radical, G(−H)^•^. Results demonstrate that 8oG formation via water addition to G^•+^ proceeds within less than the time resolution of 150 ms. The reaction of G(−H)^•^ with superoxide is more than 1000 times slower due to the formation of an intermediate with a lifetime of minutes. The overall yield of 8oG formation is low but increases considerably upon addition of dithiothreitol, thus preferring water addition over reactions depending on the presence of oxygen. Our results highlight the advantages of single-molecule real-time technology for the investigation of chemical reactions and reaction pathways. The method enables the direct observation of product formation comparable to fast kinetic methods, including flash photolysis and laser spectroscopy, but on much longer timescales. Time-resolved measurements over minutes to hours not only allow monitoring of reaction sequences that show the formation and decay of products and intermediates, but also the order of their appearance, information that is lost in classical analysis. Product yields can be obtained by counting events, in contrast to ensemble measurements that require considerable manipulation, during which intermediates may get lost.

## 1. Introduction

Organic chemical transformations usually consist of sequences of elemental reactions that lead to the formation of intermediates, often proceeding via various pathways [1]. These products are unstable and short-lived and cannot be isolated, making their discovery and characterisation difficult. Reaction mechanisms play an essential role in understanding the course of a reaction and the pathways of product formation, for which all involved intermediates must be identified. Experimentally, fast spectroscopic methods are used, or intermediates are trapped at low temperature for analysis.

In the case of cellular DNA, oxidation is the first step in a series of chemical reactions leading to the conversion of guanine, the nucleobase most easily oxidised, to products that are potentially lethal to a cell [2,3]. Therefore, it is important to understand the details of this still ongoing research spanning four decades. Due to the complexity of the reactions and the variety of products [2,4], mechanistic aspects of reaction pathways are not fully understood. Intermediates are often included by chemical intuition but have not been observed experimentally. There is agreement that type 1 photosensitised oxidation results in the formation of a G radical cation, G^•+^, and a neutral G radical, G(-H)^•^, respectively, which exist in equilibrium and react differently with water and radical species [5,6,7,8]. The state of this equilibrium and the reactivity toward nucleophiles or radicals determine the nature of products and yields and strongly depend on the chemical environment of G as a nucleoside, in ss- or dsDNA [9,10,11,12,13,14,15,16,17,18,19,20,21]. For pathway analysis, oxidation products were mainly analysed by classical methods, including separation by filtration, gel chromatography and HPLC, often in combination with MS [10,12,14,17,22,23,24], and after demanding and laborious sample preparation by hot piperidine-mediated cleavage or enzymatic digestion [9,15,16,18,19,21,25,26,27,28]. Intermediates in the type 2 photooxidation of G with singlet oxygen were investigated by trapping at low temperature followed by NMR analysis [5,29,30].

The kinetics of G oxidation was studied by Shafirovich et al. [10,12,14,17,18,28]. The group investigated reactions of G radicals with water and reactive oxygen species by time-resolved absorbance spectroscopy and subsequent product analysis by HPLC and MS. Laser and flash photolysis experiments allowed them to observe formation and decay of short-lived species on timescales ranging from ns to seconds. According to their data, in a short ss oligonucleotide, the addition of water to G^•+^ is a slow process with an estimated time constant of >3 ms (<3.3 × 10^2^ M^−1^ s^−1^) at pH 2.5 [17]. Measurements were conducted in acidic solution since, at neutral pH, G^•+^ rapidly deprotonates to the neutral radical G(−H)^•^, owing to its low pK_a_ value (3.9) [31]. In dsDNA, deprotonation proceeds via intra-base-pair proton transfer (*k*_pt_ > 3 × 10^6^ s^−1^) involving the N1 (G^•+^) and N3 (C) hydrogen bond [32]. The resulting protonated C, C(H)^+^, has a pK_a_ of 4.45, which allows calculation of an equilibrium distribution of the cation and neutral radical of approximately 30% and 70%, respectively. In dsDNA at neutral pH, the proton from both species was found to be transferred to surrounding water on the ms timescale [18]. Water addition to G^•+^ produced 8oG with a yield of only 1% [12]. As an intermediate, the water adduct radical 8-hydroxy-7,8-dehydroguanyl radical (8-HO-G(−H)^•^) was proposed but not detected. The reaction of the neutral G radical in dsDNA with superoxide was found to proceed with a rate constant of 4.7 × 10^8^ M^−1^ s^−1^ via an 8-hydroperoxide intermediate. Product analysis revealed 8oG as a minor product; the major product was 2,5-diamino-4H-imidazolone (Iz), a four-electron oxidation product [12]. When superoxide was removed by superoxide dismutase, Iz was no longer formed, and 8-oxoG became the main product [25].

In recent years, we developed a method to investigate chemical reactions in DNA immobilised in a ZMW [33,34], based on the work of the group of Korlach on single-molecule real-time (SMRT) DNA sequencing [35,36]. For this, a dye-labelled oligonucleotide was hybridised to an immobilised probe that allows the dye to come into close contact with a G:C base pair by stacking interactions. Single-molecule hybridisation was revealed by fluorescence pulses, the length and distance of which were determined by the kinetics of the process. This pulse pattern was found to change because of chemical reactions that convert the nature of the immobilised molecule and thus the hybridisation kinetics. In the specific case of oxidation of G to 8oG, a characteristic change in Cy3 fluorescence quantum yield was used as an indicator of product formation. Results were confirmed by Surface Plasmon Resonance (SPR) and static fluorescence measurements.

Here, we present the first observation of G oxidation at single-molecule resolution under conditions that allow detection of 8oG formation via different pathways, initiated by a photoinduced electron transfer from G to the dye. Two pathways can clearly be distinguished by the formation of an intermediate living on the timescale of minutes, the overall reaction rate, and the 8oG yield, which increases in the presence of dithiothreitol (DTT), a dye triplet quencher. By decreasing the rate of superoxide formation, we were able to show that the intermediate was created in the radical pathway by the reaction of the neutral G radical with superoxide. We demonstrate that the dye plays a key role in the reaction sequence leading to product formation. The conclusions from our findings are drawn based on the work of the Shafirovich group on the formation of G radicals and their reactions with water and superoxide, and the generally accepted scheme of G oxidation [3].

## 2. Results and Discussion

### 2.1. Hybridisation Properties

**Figure 1:**
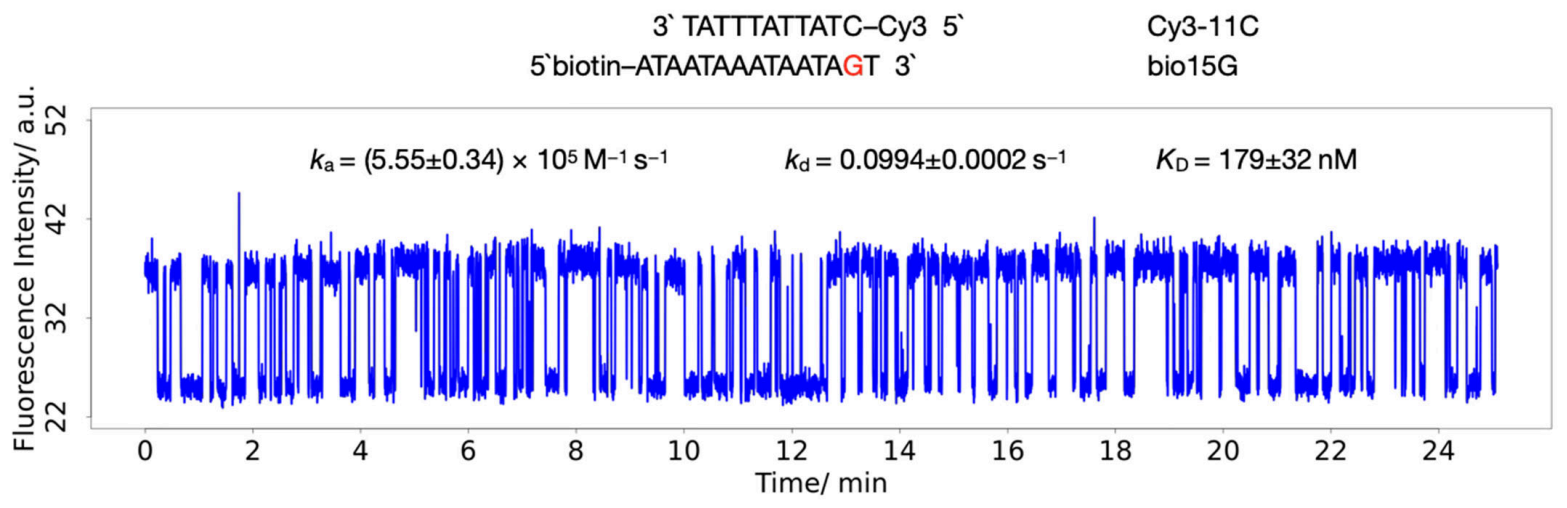
Single-molecule fluorescence trace. A continuous train of pulses arising from hybridisation of Cy3-11C to bio15G over 25 min, measured in air-equilibrated HBS. Rate constants were calculated from pulses of all 45,960 molecules within the first 100 s.

We measured single-molecule hybridisation of a short dye-labelled oligonucleotide, 5′ Cy3-CTATTATTTAT (Cy3-11C), to an immobilised probe, 5′ bio-ATAATAAATAATAGT (bio15G), in air-equilibrated HBS buffer at 20 °C in the absence and presence of dithiothreitol. DTT quenches the dye triplet state to decrease the reduction of dissolved oxygen, thus lowering the concentration of reactive superoxide [37]. The fluorescence trace in Figure 1—one out of several thousand similar traces—shows a continuous train of pulses over a period of 25 min. The pulse sequence, i.e., the length of pulses and their distance, creates a pattern that is defined by hybridisation kinetics (association and dissociation rate constant, *k*_a_ and *k*_d_), while the pulse intensity is determined by the dye fluorescence quantum yield.

For the two measurements, histograms of pulse widths (PWs) and interpulse durations (IPDs) were plotted for all 45,960 and 44,600, single molecules on the chip, respectively (Supplementary Figures S1 and S2). To calculate rate constants, more than 950,000 and 1.7 million events for both IPD and PW were used for hybridisation conducted in air-equilibrated buffer solution and in the presence of 12 mM DTT, respectively. PW and IPD are related to the rate constants of association and dissociation by IPD = 1/*k*_a_ × c and PW = 1/*k*_d_. Fitting with a two-exponential kinetics model resulted in good fits for both datasets. The rate constants *k*_a1_ and *k*_d1_ represent hybridisation kinetics, whereas *k*_a2_ and *k*_d2_ arise from products created by chemical reactions and dye effects [33]. Rate constants of association and dissociation, respectively, assigned to hybridisation and calculated from histograms for the first 100 s, were (5.55 ± 0.34) × 10^5^ M^−1^ s^−1^ and 0.0994 ± 0.0002 s^−1^ for air-equilibrated HBS, and (3.58 ± 0.13) × 10^5^ M^−1^ s^−1^ and 0.132 ± 0.001 s^−1^ in the presence of DTT (Supplementary Figure S3).

**Figure 2:**
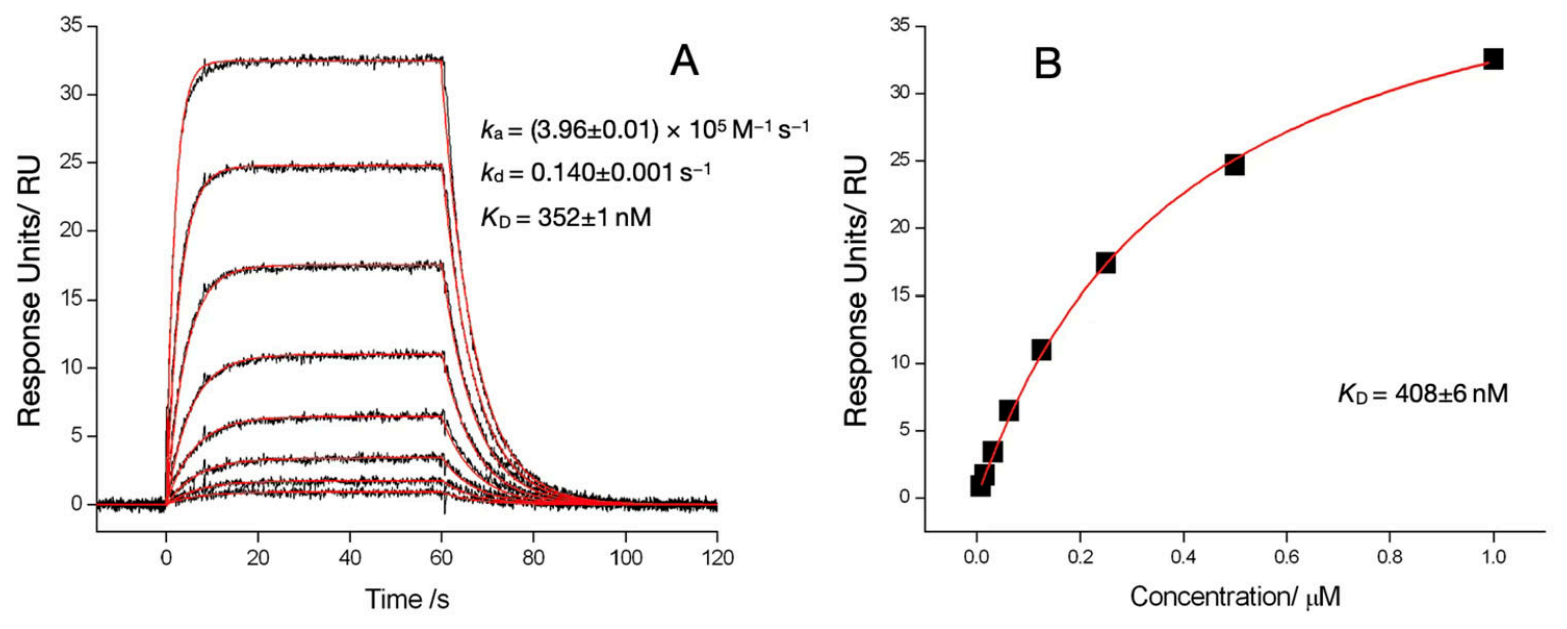
Hybridisation of CY3-11C to bio15G measured with SPR. (**A**) Sensorgrams of a dilution series of eight concentrations of CY3-11C (7.5 nM–1 µM) in HBS buffer (black lines; duplicates omitted). Red lines are the result of a global 1 + 1 fit. (**B**) Affinity plot to calculate *K*_D_ from equilibrium concentrations.

For comparison, hybridisation of CY3-11 to bio15G was measured with Surface Plasmon Resonance (SPR) under comparable conditions [33]. Sensorgrams were fitted with a 1 + 1 kinetic model (Figure 2) and are in good agreement with SPR data [33].

### 2.2. Analysis of Chemical Reactions

Single-molecule hybridisation can be analysed in two ways: at the single-molecule level by inspection of individual fluorescence traces, or by evaluation of pulses and interpulse durations in sum for the whole chip. The latter provides an overview by calculating rate constants from histograms of PW and IPD, respectively, for all immobilised single molecules over 25 min (Supplementary Figures S1 and S2) and for short time intervals of 100 s. Time interval analysis was introduced as a method in a previous publication [34].

In part 1 of this series on single-molecule chemistry, we demonstrated that, in oligonucleotide hybrids, excitation of the labelling dye at 532 nm triggers photoinduced electron transfer that converts G to various reaction products [34]. These products tend to destabilise the hybrid, leading to a change in hybridisation kinetics and/or dye fluorescence. To identify reaction products, the pulse patterns created were compared with those of synthetic standards in single-molecule real-time, SPR and fluorescence measurements. Depending on the oligonucleotide design, chemical reactions were found to proceed in both strands to oxidise G and AAA (triple A) (Supplementary Figures S4 and S5). Whereas, after dissociation, the analyte escapes into the bulk solution, the immobilised probe allows investigation of chemical reactions by means of changes in hybridisation kinetics and dye fluorescence over a long period of time.

Product yield analysis revealed that changes in hybridisation kinetics mainly arise from the formation of reaction products in the probe, characterised by a 10-fold or greater decrease in affinity of the dye-labelled analyte. An exception is the formation of 8oG, which shows similar hybridisation kinetics but a unique decrease in fluorescence intensity. The conversion of G to 8oG creates a pulse pattern that is easy to identify [34,38].

Since the most stable hybrid arises from the perfect match configuration, a chemical transformation of a nucleobase decreases hybrid stability depending on the nature of the lesion created, and its position within the hybrid, similar to the effect of a mismatch [39]. In a single-molecule measurement, upon hybridisation with Cy3-11C, this leads to pulse pattern changes from long (bio15G) to shorter pulses (bio15X, X being the reaction product). In our model system, this was found for oxidation products, including Gh, Sp and Iz, which behave like mismatches, in agreements with SPR measurements using reference compounds (unpublished data). In a short oligonucleotide hybrid, these lesions are characterised by a strong decrease in affinity, mainly caused by an increase in *k*_d_ by an order of magnitude and a slower association. The formation of 8oG reduces the affinity by a factor of about two, creating a pulse sequence similar to that of G (see Supplement of [40]).

For low-affinity hybrids with *k*_d_ >> 1 s^−1^, the limited time resolution of our method does not allow us to detect hybridisation. Strong destabilisation results from conversion of nucleotides at positions closer to the middle of the hybrid [39], and, at a terminal position, from reaction products that do not stabilise the hybrid by hydrogen bonding and/or stacking interactions with a base pair and the dye. Reaction products at the detection limit produce ill-defined and irregular pulse sequences that cannot be evaluated.

#### 2.2.1. Dye Effects

**Scheme 1:**
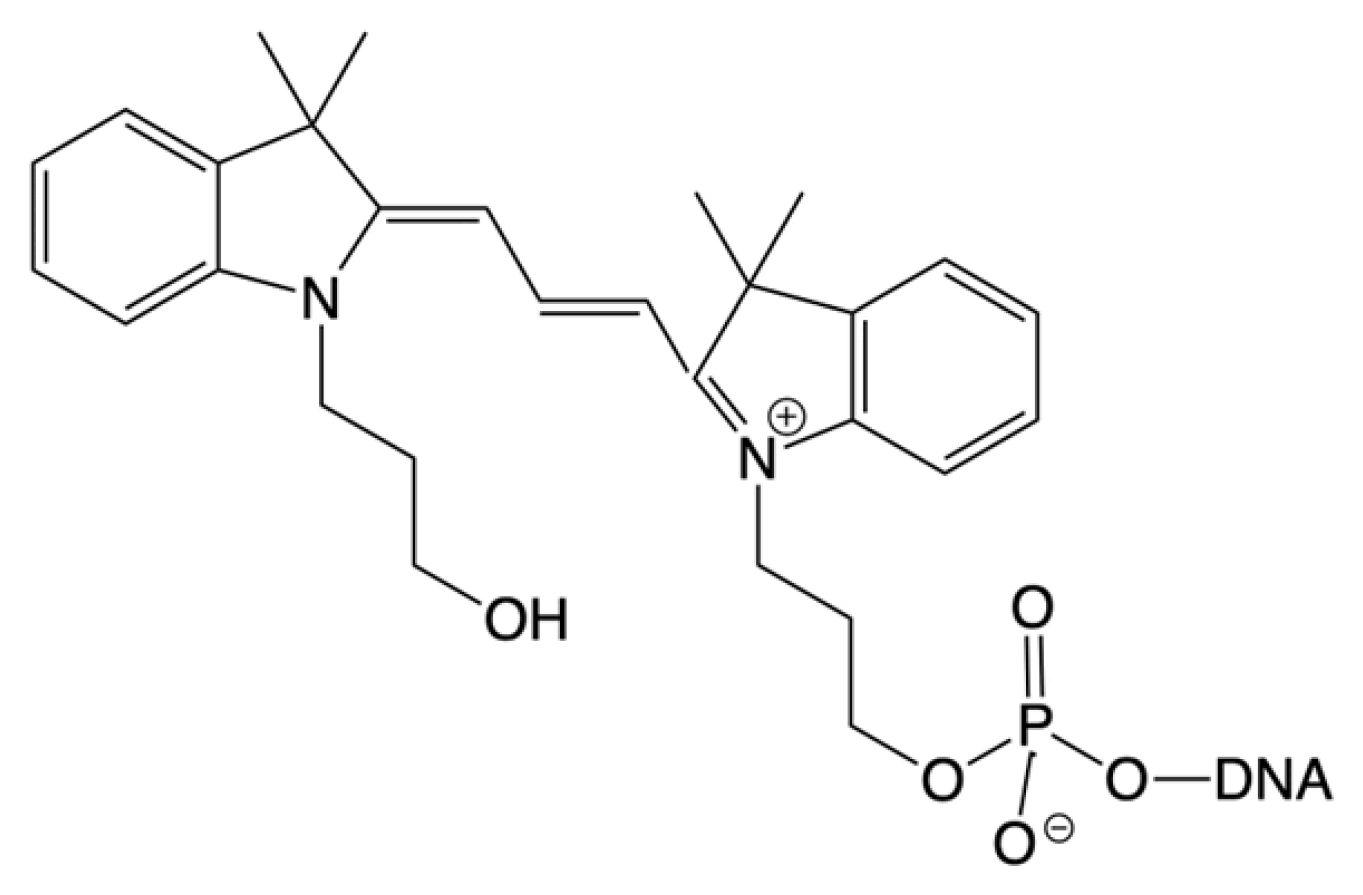
Chemical structure of Cy3 linked to the 11 nt oligonucleotide via the 5′ phosphate group.

The labelling dye plays the pivotal role in the ongoing processes. The fluorescence quantum yield of many cyanine dyes is low (Cy3 in PBS buffer: 0.04) but increases upon interactions that block the trans-cis isomerisation of exocyclic double bonds [41]. This effect can be exploited to investigate inter- and intramolecular interactions and to discriminate between base pairs converted by chemical reactions [34,42,43,44]. In our setup, the dye was attached to the 5′-end of the 11 nt analyte, Cy3-11C, via a short linker (Scheme 1). In a hybrid with bio15G, this allows stacking interactions with the G:C base pair, bringing the dye into close contact with the G donor. In this conformation, photoinduced electron transfer—the initial step of product formation—is most likely.

In the hybrid, Cy3 has three basic functions. Firstly, it acts as an electron acceptor that oxidises G by photoinduced electron transfer. In a second step, in competition with fast back electron transfer, the reduced dye transfers the electron to dissolved oxygen. This produces a long-distance—and long-lived—charge-separated state as a precondition for the oxidised G to react with water and reactive oxygen species.

Secondly, the dye in its excited state reduces oxygen [45,46], which is the main source of superoxide, O_2_^−•^, as the agent that reacts with the neutral G radical, G(−H)^•^ [12,47,48]. Reactions typically proceed in the triplet excited state, ^3^Cy3, since the lifetime of the singlet state is too short for bimolecular reactions to be efficient at low reactant concentration [42]. Cy3 and its derivatives are relatively stable to superoxide and singlet oxygen, as demonstrated by the fact that fluorescence measurements are generally conducted in non-degassed solutions. The photostability of various unsymmetrical cyanines conjugated to DNA was investigated by Schwechheimer et al. Under conditions of constant irradiation, measurements revealed half-lifetimes of 0.5–1 h [38], which is two orders of magnitude longer than the average time a dye molecule was irradiated in our experiments (mean PW ca. 10 s), excluding dye degradation as a major process. This is in line with the observation of long pulses in SMRT experiments using Cy3-labelled 8 nt oligonucleotides that can extend over 10 min or longer (Supplementary Figure S6). The addition of DTT inhibits superoxide formation due to quenching of ^3^Cy3 by an electron transfer mechanism [37], which decreases the rates of radical reactions depending on the superoxide concentration. Further, quenching of ^3^Cy3 competes with electron transfer from G, which is the reason for the reduced reactivity in the presence of DTT.

Thirdly, when stacking with the base pair and thus being as close as 0.5 nm to the scene of events, the dye reveals chemical processes within the base pair by emission changes [40,49]. This allows detection of changes in electron density distribution and/or the spatial re-arrangement of atoms within the stacking base pair in case of formation of 8oG and Sp, respectively. Thus, reaction products can be discriminated, in addition to their effect on hybridisation kinetics. Fluorescence intensity changes were impressively demonstrated in long-lived intermediates observed during oxidation of 8oG (Supplementary Figure S3). It is important to note that dye fluorescence is only sensitive to processes within the stacking base pair and the next overhang nucleotide.

Many dyes are known to produce singlet oxygen, ^1^O_2_, which preferentially reacts in type II cycloadditions with G. In a recent work, one of us (AF) investigated the oxidation of guanosine and a G4 quadruplex by free dyes, including (di-sulfonated) sulfoCy3 [19]. It was demonstrated that, under bimolecular conditions, sulfoCy3 does not react by electron transfer, since no typical type I photooxidation products, Iz and Z, were formed. Instead, singlet oxygen was produced at a very low rate. ^1^O_2_ is known to oxidise guanosine and G4 by a [2 + 4] cycloaddition to 8oG and higher oxidation products, including Sp [5,11,50,51]. For a dye covalently linked to DNA, conditions are fundamentally different, since within the hybrid, the donor (G:C base pair)–acceptor (Cy3) distance is reduced to about 0.5 nm due to stacking with the base pair [49]. In a bimolecular experiment, this short distance would only be achieved at molar dye concentrations. Owing to the strong distance dependence, an affinity of the dye to the base pair and sufficient free energy are preconditions for photoinduced electron transfer. For Cy3 and its derivatives, type II photooxidation—at low yield—can only be observed when electron transfer is not efficient. The low rate of ^1^O_2_ production is a major reason for the stability of Cy3 in our experiments.

The dye dependence of product formation and yields results from a complex interplay of electronic and steric factors that have not been investigated systematically. Qualitatively, the overall product yield decreases with decreasing affinity of the dye for the base pair, a phenomenon described by the van′t Hoff stabilisation energy [40]. With increasing negative charge of the dye, in the order Cy3 (+1) > Cy3B (0) > DY-547 (−1) > DY-549 (−3), the probability to dwell in a stacking conformation is lower, as shown by the weight of the long-lifetime components in time-resolved fluorescence measurements (unpublished data). In non-stacking conformations, excitation energy is primarily dissipated through trans-cis isomerisation. This reduces the rate of photoinduced electron transfer as the first step of the reaction sequence leading to the formation of G^•+^ and, subsequently, reaction products. Furthermore, the reduction potential is expected to decrease with the number of negative charges, thereby reducing the free energy of electron transfer to the dye and, consequently, the probability of photoinduced electron transfer. For the same reason, the ability of the dye excited state to reduce oxygen is expected to increase with negative dye charge. In a stacking conformation, the dye partly shields the base pair from attack by reactants, thereby limiting access to the nucleotides and thus their reactivity. This effect proved to be decisive when reactants such as superoxide were generated not by reduction via the dye but by an external source. Based on the observation that more fluorescence traces exhibiting a continuous pulse sequence (Figure 1) were found along the above dye series, the rate-limiting step of all processes appears to be the second electron transfer step leading to the formation a long-distance charge-separated state by formation of superoxide. The initial electron transfer can be controlled by the dye reduction potential and the degree of stacking interactions with the nucleobases, as determined by the dye charge [40]. In addition to the superoxide concentration, the state of equilibrium between G^•+^ and G(−H)^•^ determines the yield of reaction products. This equilibrium can be controlled by substituents at the nucleobase and by mismatch base pairs. In summary, by selecting a suitable labelling dye, an oligonucleotide design and additives including a triplet quencher and oxygen reduction systems, it is possible to control the outcome of an experiment in such a way that the formation of oxidation products is maximised or prevented. Based on this premise, DY-549 is the dye of choice to serve as a mostly inert fluorescence label and to achieve the greatest effect when attached to G.

An important advantage of our single-molecule setup is the exclusion of molecular interactions with neighbouring probes and bulk analyte [33,52,53,54]. In a ZMW, due to the confinement of the excitation volume to some attoliters [55], the fluorescence background is strongly reduced. At nM concentration, only one dye-labelled analyte molecule is irradiated, limited by the time the hybrid exists. In contrast to classical photochemical experiments, this arrangement prevents the bulk solution from irradiation and enrichment of photodegradation products.

#### 2.2.2. Oxidation of G to 8oG: Pathway Analysis

**Figure 3:**
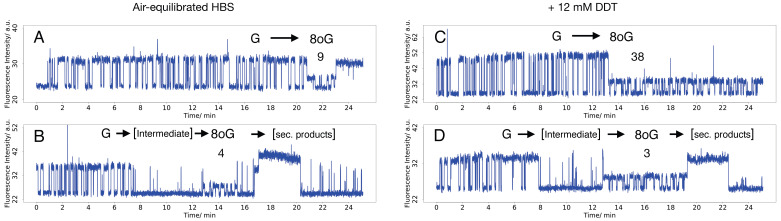
Pathway analysis. Fluorescence traces of hybridisation of Cy3-11C to bio15G in air-equilibrated HBS (**A**,**B**) and in the presence of 12 mM DTT (**C**,**D**). Oxidation of G to 8oG is revealed by a characteristic decrease in fluorescence intensity with virtually no change in hybridisation kinetics, i.e., the pulse sequence proceeds with very similar PW and IPD. Fluorescence traces demonstrate that the formation of 8oG proceeds via different pathways: (**A**,**C**) fast creation of 8oG proceeding within the time resolution of 150 ms; (**B**,**D**) reaction of G to 8oG through an intermediate having a lifetime of ca. 5 min. 8oG is less stable to oxidation and converts within minutes to secondary products. The number of events found in 1090 randomly selected fluorescence traces is given.

The starting point is a fluorescence trace of hybridisation of Cy3-11C to bio15G, characterised by a continuous train of pulses over 25 min without formation of products (Figure 1). About 3 out of 100 molecules show behaviour depicted in Figure 3A. In this trace, after ca. 21 min, the fluorescence intensity drops by 60% with no apparent change in hybridisation kinetics. Since a pulse pattern was defined by the combination of PW, IPD and fluorescence intensity, a pattern change indicates the formation of a new species.

**Figure 4:**
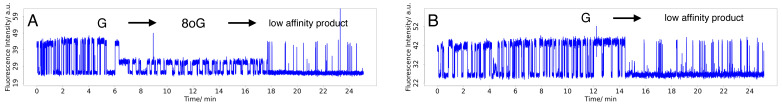
Fluorescence traces of hybridisation of Cy3-11C to bio15G (air-equilibrated HBS, DTT) showing the conversion of G to a product of low affinity: (**A**) via 8oG [pathway (1) in Scheme 2], and (**B**) without intermediate formation of 8oG [pathway (3)].

In case of Figure 3A,C, the pattern change is the signature of 8oG formation. This assignment is based on four observations presented in [34]. In short, static fluorescence spectra using synthetic standards reveal quenching by 60% when Cy3 is stacking with a C:G and C:8oG base pair, respectively. Hybridisation measured with SPR showed that 8oG substitution does not significantly destabilise a hybrid, resulting in similar hybridisation kinetics (Supplement of [40]), in contrast to other lesions we investigated by means of standards. Furthermore, when using an 8oG-modified probe, a pattern with the same characteristics was observed. Finally, since the oxidation potential of 8oG is 0.55 eV lower than that of G [56], oxidation is much faster, which leads to a pulse sequence that changes within minutes to indicate secondary product formation. We also demonstrated that additives, including triplet quencher (DTT, Trolox) and an oxygen reduction system (protocatechuate 3.4-dioxygenase/protocatechuic acid, PCD/PCA), do not affect hybridisation [33,34] but influence the reactivity of the dye and product formation. The characteristics of 8oG formation is irreversible and clearly stands out from all other patterns observed in hundreds of experiments. This unique pattern allows identification of pathways of G oxidation proceeding via 8oG as an intermediate, or direct reaction to higher oxidation products, including Gh, Sp, and Iz (Figure 4A,B). An important aspect of the fluorescence traces in Figure 3A,C is the time it takes G to convert to 8oG. The time resolution of 150 ms sets an upper limit for the sequence of reactions that create 8oG via this pathway.

Likewise, in fluorescence traces in Figure 3B,D, the pulse patterns of G and 8oG, respectively, are separated by a series of short pulses of non-uniform intensity characteristic of a hybrid of low affinity. We suggest that this new pattern arises from hybridisation of Cy3-11C to a species in which G was converted to a reaction product. This intermediate product strongly destabilises the hybrid to a similar order of magnitude as the effect of a mismatch. The change in fluorescence intensity indicates that the reaction must have proceeded at G. Our argumentation is further supported by the fact that addition of DTT decreases the yield of its formation by ca. 25% (Table 1), an effect that can hardly be explained by conformational changes within the hybrid or dye stacking. The intermediate exclusively converts to 8oG, since no other products were observed. Under the assumption that each pulse pattern change arises from a different species created by chemical reactions or structural changes within the hybrid, including equilibrium between species, sequences of reactions can be monitored (examples are given below, Figure 6).

**Scheme 2:**
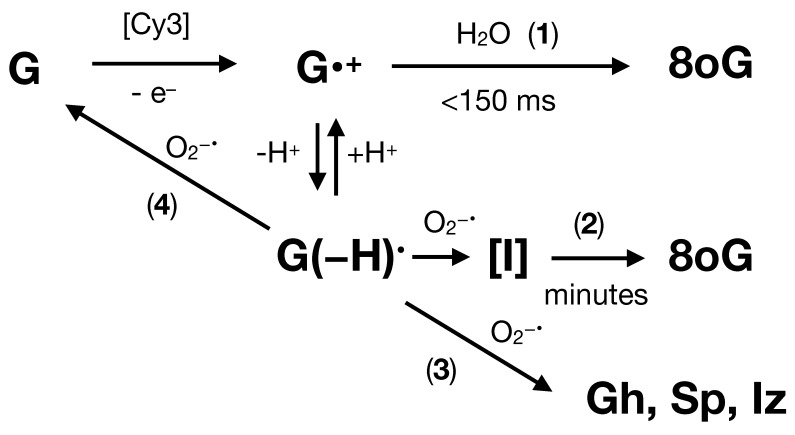
Simplified reaction scheme for the oxidation of G via electron transfer. The intermediate [I] observed in Figure 3B,D is created within the radical pathway.

**Figure 5:**
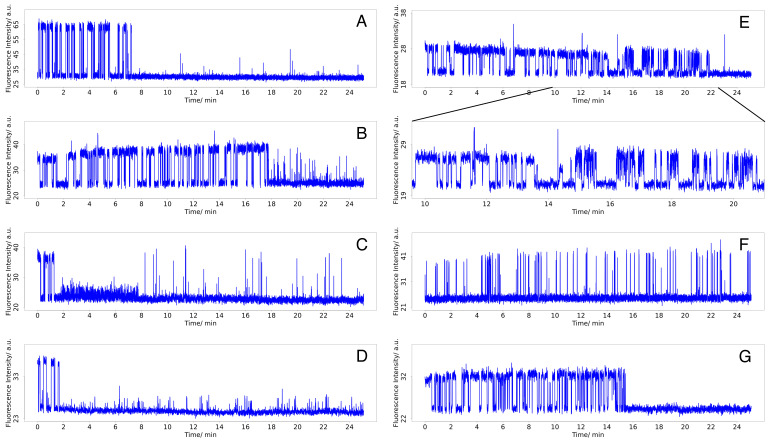
Fluorescence traces of hybridisation of Cy3-11C to bio15G that cannot be evaluated. (**A**) Product formation results in a pattern that is not analysable. (**B**–**F**) Unidentified products. The pulse pattern cannot be assigned owing to a lack of reference compound. (**C**,**D**) Formation of products strongly quenching fluorescence. (**E**) Formation of a product that undergoes reversible reactions. (**F**) Although the pulse pattern is well defined, it cannot be assigned to a (low-affinity) product since there is no pattern of the parent compound. The intensity change relative bio15G cannot be determined. The reason may be a chemical reaction during the preparation time of the RSII before the measurement was started (ca. 90 s), or an impurity in bio15G. (**G**) Termination. A product is created having no affinity to Cy3-11C.

To allocate the pattern changes in Figure 3 to reaction pathways in Scheme 2, measurements conducted in the absence and presence of DTT were compared with respect to the number of events as a measure of product yield. To this end, the pulse patterns of a large number (1090) of randomly selected fluorescence traces, sufficient for statistical analyses, were analysed by counting the number of traces comprising a specific feature. This procedure involved assignment of the characteristic pattern change of 8oG formation (Figure 3 and Figure 4A), the formation of low-affinity products characterised by short-pulse sequences (Figure 4A,B), and products revealed by ill-defined patterns and terminations (Figure 5). In reaction sequences, the first product created was counted. For example, Figure 4A was annotated as 8oG formation, whereas traces including Figure 4B are counted as low-affinity products.

For measurements in the absence and presence of DTT, similar pulse patterns were found, but product yields were significantly different. Most evidently, in the presence of DTT, there were more than twice as many fluorescence traces featuring an unaltered pulse sequence (without DTT: 70/1090 traces; with DTT: 155/1090 traces). In contrast, the number of pattern changes to shorter pulses, indicating the formation of low-affinity products including Sp, Gh, and Iz, was reduced by a factor of nearly 5 (43/1090 vs. 9/1090), in line with the consumption of oxygen for these products to be formed [12,16,28]. The formation of 8oG via the intermediate (Figure 3B,D) was rare in both the absence and presence of DTT and showed similar yields (4/1090 vs. 3/1090). However, in the presence of DTT, there were four times more traces featuring 8oG formation (9/1090 vs. 38/1090) without intermediate (Figure 3A,C). Remarkably, DTT significantly reduced the number of fluorescence traces containing ill-defined pulse patterns arising from unidentified reaction products with very short hybrid lifetimes (53/1090 vs. 8/1090) and other not identifiable products (111/1090 vs. 66/1090) (Figure 5).

For better statistics, 3500 randomly selected trace files were screened for 8oG formation. In the absence and presence of DTT, fifteen and nine fluorescence traces, respectively, were found to show the intermediate pathway. Including the number of ZMWs containing a single molecule, reaction yields for the different processes were calculated (Table 1). Results confirm that, upon addition of DTT, reduced superoxide production decreases 8oG formation via the intermediate and other reactions that depend on superoxide concentration [pathways (2,3,4) in Scheme 2]. This lead us to conclude that fluorescence traces featuring the intermediate (3B,D) show 8oG formation via the radical pathway.

This result was surprising in two ways: Firstly, the yields of 8oG formation via the two pathways are similar. This was unexpected considering the rate constants for the addition of water and superoxide to G^•+^ (<3 × 10^2^ M^−1^ s^−1^) and G(−H)^•^ (4.7 × 10^8^ M^−1^ s^−1^), respectively, which differ by six orders of magnitude [12,17]. The reason for this is the difference in the concentration of the reactants, which for the latter is lower by a similar degree. In air-equilibrated solution, the oxygen concentration is approximately 250 µM. In flash photolysis experiments, the superoxide concentration was determined to be ca. 1 µM [12], 55 × 10^6^ times lower than the water concentration. This explains why more 8oG was produced by water addition and the increased yield upon addition of DTT.

Secondly, although the elemental reaction with superoxide is faster, the radical pathway to 8oG is at least 1000 times slower, delayed by the formation of the intermediate. Water addition to G^•+^ proceeds without a detectable intermediate within 150 ms. The formation of a transient 8-hydroxy-7,8-dihydropurin-7-yl radical was discussed but does not appear in our measurements [6,26,31].

Our findings are in line with the results of other groups. Kasai et al. investigated the oxidation of G to 8oG in calf thymus DNA using riboflavin as a photosensitizer [26]. Expecting to find type II photooxidation, the authors did not observe an increase of 8oG yield in D_2_O as an indication of ^1^O_2_ to be involved. As an explanation, an electron transfer mechanism was suggested to create the G^•+^ cation. In dsDNA, processes following the primary electron transfer to riboflavin as an acceptor are similar to those in Cy3-labelled hybrids. A fivefold enhancement of 8oG formation was found when the reaction was conducted in oxygen-free solution, which is in good agreement with the fourfold increase in our experiments. Similarly, a fourfold increase was measured by Misiaszek et al. when oxidation was performed in Ar-purged solution [12]. Since ^16^O_2_ and H_2_^18^O were used, the experiments proved the additional oxygen atom in 8oG to originate from oxygen and water when conducted in the presence and absence of oxygen, respectively. Consistently, the overall yield of 8oG formation was found to be low, typically in the range of a few percent [2,12,26,57,58].

**Figure 6:**
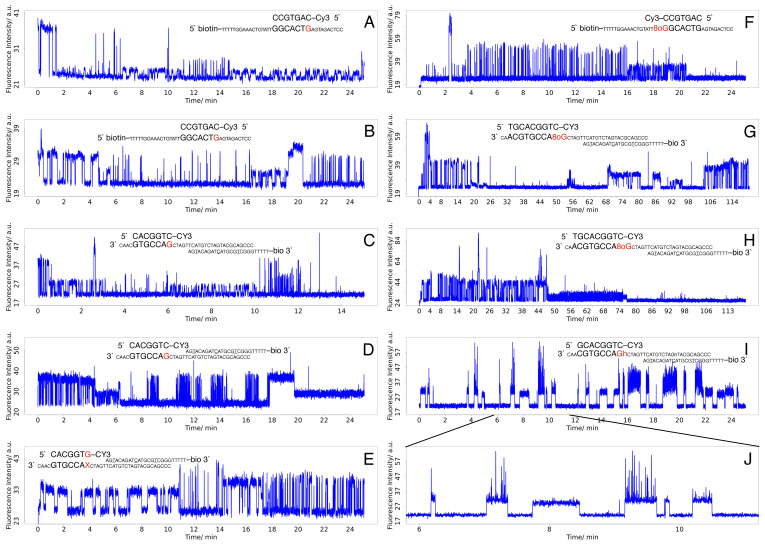
Examples of fluorescence traces demonstrating the potential of single-molecule pathway analysis in 7 nt and 8 nt hybrids of different sequence context and with modified bases (8oG, Gh). The original system is shown in the image; the nucleotide to react is marked in red. Each pulse pattern represents a reaction product or an intermediate. Fluorescence traces show sequences of chemical reactions resulting in a remarkable number of different products. Most of these products cannot be identified owing to the lack of synthetic standards. (**A**–**E**) Conversion of G to products, including 8oG (**B**–**D**), followed by secondary reactions. (**B**) The large pulse at 19 min indicates the conversion of 8oG via two intermediates that are revealed by different fluorescence intensities within this pulse. (**C**) There are seven different pulse patterns; only the first oxidation product (8oG) can be assigned. (**D**) Oxidation of G to 8oG, which converts after 8 min to a low-affinity product that exists for ca. 1 min. There are four (five?) groups of similar pulses, which suggests the existence of a reversible reaction. (**F**–**H**) Originating from an 8oG-modified probe, 8oG converts to various products, in (**G**,**H**) monitored over 120 min. (**I**,**J**) Reversible conversion of Gh (to iminoallantoin?) in a Gh-modified standard. Cy3-labelled 8 nt oligonucleotides were used (**G**–**I**) to increase the lifetime of the hybrid.

In this context it should be noted that product yields are only partially comparable to literature data. Counting the number of conversions at the single-molecule level allows determination of precise yields. In contrast, classical analysis of products prone to secondary reactions, including 8oG, only yields stationary concentrations [29], which are lower unless the reaction is quenched. Furthermore, there are inevitable losses during sample processing. Under conditions of an SMRT experiment, secondary reactions in the presence of oxygen invariably convert 8oG to higher oxidation products within minutes. It is an advantage of the method to show the progression of chemical reactions by means of the order of formation and decay of products in real-time (Figure 6), and whether formation of higher oxidation products proceeds from G or via 8oG as an intermediate (Figure 4).

Singlet oxygen, produced by triplet–triplet annihilation (^3^dye and ^3^O_2_) or radical recombination (G(−H)^•^ + O_2_^−•^) [pathway (4) in Scheme 2] [25], opens a reaction pathway that does not depend on electron transfer [2,5,19,30,46,59,60]. Type II photooxidation proceeds by [2 + 4] cycloaddition of ^1^O_2_ to G via endoperoxide and hydroperoxide intermediates. In a single-molecule measurement, fast oxidation with ^1^O_2_ would be revealed by a pulse pattern change similar—and indistinguishable—to that shown in Figure 3A. The observed increase of 8oG yield in the presence of DTT contradicts a contribution of ^1^O_2_, since it is efficiently quenched by DTT [61]. Given the low rate of ^1^O_2_ production [19], cycloadditions are very rare events, if they proceed at all, which also accounts for the remarkable stability of Cy3 under our experimental conditions.

As demonstrated by the pulse pattern changes shown in Figure 3A,B,D, Figure 4A, and Figure 6B–D,F–H, 8oG is highly reactive under oxidising conditions and converts within minutes to a larger number of oxidation products [9,56,62,63]. Although the effect cannot be quantified, inspection of many fluorescence traces indicates that pulse sequences of 8oG are longer in the presence of DTT. Quenching of the dye triplet state reduces both the probability of photoinduced electron transfer and the reduction of oxygen to superoxide, leading to an increased lifetime of 8oG. Fluorescence traces in Figure 3A,B,D show that 8oG oxidation often proceeds via intermediates characterised by long and, in some cases, structured pulses that exist on the timescale of minutes (Figure 6B, Supplementary Figure S3). The oxidation of 8oG will be discussed in a forthcoming publication.

Single-molecule experiments in ZMW nanostructures are a unique method to study chemical reactions in real time over periods of hours. Fluorescence traces allow deep insight into the details of chemical reactions, including the formation of short-lived intermediates, equilibria, and product lifetimes. Although the time resolution is limited to 150 ms, reaction products existing on the timescale of seconds and longer can be detected in the order of their formation, as demonstrated through illustrative examples in Figure 6.

An apparent limitation of our method is the fact that it does not reveal the nature of the product behind a specific pulse pattern. As in identification by gel chromatography or HPLC, reference compounds are needed to determine hybridisation kinetics and, in addition, the fluorescence properties of the dye when stacked with the lesion-modified base pair [34]. Therefore, it is not possible to identify intermediates and products that cannot be produced in substance. However, we succeeded in the detection and characterisation of Iz by hybridising Cy3-11C to a modified bio15G in which G was substituted by 8-methoxydeoxyguanosine, 8-MeO-G [64]. We demonstrated that Cy3 oxidises 8-MeO-G, which allowed us to study this complex reaction at the single-molecule level. The oxidation product was sufficiently stable to be characterised with SPR, fluorescence spectroscopy, and MS (unpublished data). Recently, Lu et al. developed a kinetic method that combines in situ formation of ^1^O_2_ and spectroscopic analysis of G oxidation products by absorbance, fluorescence, and mass spectroscopy, which seems to be the method of choice for this purpose [24].

Therefore, we can only speculate about the nature of the observed intermediate. According to the literature, the neutral G radical is assumed to react with a superoxide radical at positions 5 and 8, the latter yielding 8oG (Scheme 2) [2,12,14,28,65]. According to Misiaszek et al., superoxide addition at position 8 yields 8-hydroperoxide-G [12], which is an accepted intermediate in the oxidative pathway with ^1^O_2_ [11,24,29,30,65,66]. However, the properties of the intermediate derived from fluorescence traces contradict this assumption, and 8-hydroperoxide-G is too short-lived to be detected at room temperature [29]. An alternative explanation is the reversible formation of a radical adduct at the C4 or C5 position that produces an sp^3^-hybridised carbon atom. The distortion of the flat ring system diminishes stacking interactions with adjacent nucleobases and the dye, thus destabilising the hybrid, which is in line with the observed low affinity.

## 3. Materials and Methods

All experiments were conducted in HBS buffer (Teknova, CA, USA), containing 10 mM HEPES, 150 mM NaCl, 3 mM EDTA, and 0.05% Tween-20, at 20 °C. Single-molecule hybridisation of a dye-labelled 11 nt oligonucleotide, Cy3-11C (5′ Cy3-CTATTATTTAT), to an immobilised reverse-complimentary 15 nt oligonucleotide, bio15G (5′ bio-ATAATAAATAATAGT), was measured in a commercial DNA sequencer (RSII), which is a confocal microscope (Pacific Biosciences, Menlo Park, CA, USA) [33,35,36]. For comparison of kinetic data, SPR measurements were conducted using a Biacore T200 instrument (Cytiva, Freiburg, Germany). Bio15G was designed to contain a single G to limit charge transfer and secondary reactions at distant sites within the hybrid. To obtain a sufficiently stable hybrid, an 11 nt oligonucleotide was chosen, having a calculated melting temperature of room temperature, similar to 7mers used in earlier studies [33,34]. The labelling dye, Cy3, was attached to the 5′ end to allow stacking with the G:C base pair (Scheme 1). HPLC-purified oligonucleotides were obtained from Microsynth (Balgach, Switzerland).

Details of the experimental setup of SMRT measurements were described in a previous publication [33]. Experiments were conducted in an SMRT chip (Pacific Biosciences) that contains >163,000 zero-mode waveguide (ZMW) nanophotonic confinement structures of ca. 150 nm in diameter and height. Fluorescence was excited at 532 nm and detected with an electron-multiplication charge-coupled device (EM-CCD) array [36]. Data were collected at 75 Hz for plotting fluorescence traces, 11 data points were averaged, giving rise to a time resolution of 150 ms.

For an experiment, nanostructures were first coated with 1.5 µM streptavidin (Sigma/Merck, Buchs, Switzerland) for 300 s, then bio15G at 130 pM was immobilised for 90s in freshly prepared HBS buffer diluted from 20× stock. Measurements were conducted for 25 min at an analyte concentration of 200 nM in HBS without dye protection and in the absence of oxygen scavenger. For comparison, 12 mM DTT (Sigma/Merck) was added. DTT is known to quench ^3^Cy3 and ^1^O_2_ [60], but not to react with superoxide, O_2_^−•^ [12,60]. Chips were regenerated by adding DMSO for 1 min to remove immobilised streptavidin and bio15G, followed by washing steps with water and HBS buffer, and were used up to four times.

Measurements repeated on a second chip show identical pattern changes. Additionally, we conducted experiments with longer analytes (12 nt, 13 nt) to stabilise the hybrid, and with mismatch oligonucleotides (Cy3-12G, Cy3-12A) to allow the dye to stack with GG and AG mismatch base pairs, respectively. These measurements support our interpretation of the data.

Data were evaluated with in-house made software to determine the number of molecules immobilised in a nanostructure and to calculate global kinetic constants, *k*_d_ and *k*_a_, from PWs and IPDs of ZMWs having one molecule. For this, histograms of PWs and IPDs were fitted with a 1 + 1 and a sum of two-exponential kinetic model. Additionally, several thousand fluorescence traces were evaluated by visual inspection and annotated for characteristic features and pulse pattern changes.

For the two measurements presented here, 45,960 and 44,600 ZMWs with a single molecule of bio15G were found. Hybridisation with Cy3-11C produced a large number of fluorescence traces suitable for pathway analysis and for quantitative evaluation of PW and IPD. The large number of events gives rise to excellent statistics for calculation of rate constants.

Surface Plasmon Resonance was measured with a Biacore T200 instrument on a CMTEG chip (Xantec, Düsseldorf, Germany). The chip was first coated with streptavidin using our standard protocol [33]. Bio15G at 20 nM was immobilised for 30 s to a surface density of 84 RU. A dilution series of eight concentrations of Cy3-11C (7.8–1000 nM) was injected in duplicate in HBS buffer for 60 s to reach equilibrium. Dissociation of the hybrid was finished after 60 s. An empty flow cell was used as reference; additionally, five injections of buffer were averaged and subtracted (double referencing). Sensorgrams were evaluated with BiaEvaluate 4.1 software (Cytiva) by fitting the data with a 1 + 1 kinetic model to obtain kinetic constants (*k*_a_, *k*_d_) and the equilibrium constant, *K*_D_. For an affinity plot, intensities at equilibrium (plateau values) were plotted vs. concentration.

## 4. Conclusions

We demonstrated that, in single-molecule hybridisation of a Cy3-labelled analyte, oxidation of G to 8oG can be detected in the oligonucleotide hybrid, and that dye-sensitised chemical reactions proceed via different pathways. Our results suggest that 8oG, created by addition of water to G^•+^, appears within the time resolution of 150 ms. The reaction of the neutral G radical with superoxide leads to the formation of an intermediate that converts to 8oG within minutes. Therefore, the yield of reaction products strongly depends on the superoxide concentration and the state of equilibrium between G^•+^ and G(−H)^•^. The oxidised G reacts as an electrophilic species or a radical to 8oG and higher oxidation products via different pathways.

In the absence of DTT, the 8oG yield is low (3%), whereas the main oxidation products are Gh, Sp, and Iz (14%), which require superoxide for their formation. At low superoxide concentration, the ratio of formation of 8oG (12%) and higher oxidation products (3%) is reversed.

The intermediate we found is difficult to detect with other methods commonly used and has obscured detection because of its low yield and the lifetime, which is too short for analysis by HPLC/MS and too long to be observed in flash photolysis experiments. Due to lack of a synthetic standard, it is not possible to confirm the nature of this intermediate.

The dye plays a central role as oxidant to create G^•+^ and as a reductant to transfer the electron to dissolved oxygen. Upon addition of DTT, ^3^Cy3 is quenched, inhibiting the formation of the charge-separated state and reducing superoxide formation. The overall yield of oxidation products can be controlled by the charge of the dye. With increasing negative dye charge, electron transfer to create G^•+^ becomes less likely, and the yield of product formation decreases. This can be used in experiments when typical stabilising agents such as triplet quencher and oxygen scavenger cannot be added.

Single-molecule real-time experiments are an excellent way to study chemical reactions in oligonucleotides that form products on the timescale of seconds and minutes. Although elemental reactions proceed on much faster scale, product formation can be followed with a time resolution of 150 ms. If a product or an intermediate exists for seconds or minutes, it can be detected by a change in the pulse pattern and/or the fluorescence of the labelling dye. However, for identification, a reference compound is needed to compare hybridisation and dye fluorescence properties with SPR and static fluorescence spectroscopy, respectively, which, for short-lived intermediates, is hardly possible.

## Figures and Tables

**Table 1:** Yields of G oxidation products calculated from numbers of characteristic features in fluorescence traces. Standard deviations are calculated by taking the square root of the number of events (Poisson-distributed) and are given in paratheses.

Reaction Product	HBS Buffer	+DTT
No reaction	0.229 ± 0.030	0.520 ± 0.042
8oG without intermediate ^1^	0.029 ± 0.01	0.128 ± 0.021
8oG via intermediate ^2^	0.013 ± 0.006	0.009 ± 0.006
Short-pulse sequences (Sp, Gh, Iz)	0.140 ± 0.022	0.031 ± 0.01
Undefined products Ill-defined patterns	0.364 ± 0.035 0.173 ± 0.024	0.165 ± 0.027 0.027 ± 0.09
Terminations	0.052 ± 0.013	0.060 ± 0.014

^1^ Figure 3A,C; ^2^ Figure 3B,D.

## Data Availability

Inquiries can be directed to the corresponding author.

## Supplementary Materials

The following supporting information can be downloaded at: https://www.mdpi.com/article/10.3390/molecules31101564/s1, Figure S1: Histogram plots for hybridisation of 200 nM Cy3-11C to bio15G in air equilibrated HBS over 25 min. SPR data demonstrate that hybridiation is a first-order process (Figure 2). Under SMRT conditions, a single-exponential kinetic model does not fit the data well (black line) [33]. A biexponential model is a very good approximation (blue line) indicating the existence of an additional process (or processes) (*k*_d2_, *k*_a2_) which were identified as chemical reactions changing the nature of the parent compound and thus hybridisation kinetics. For comparison with SPR data, histograms from the first 100 s were fitted (results in Figure 1 and Figure S3) to limit the effect of chemical reactions. Figure S2: Histogram plots for hybridisation of 200 nM Cy3-11C to bio15G in air equilibrated HBS/12 mM DTT over 25 min. Single exponential and biexponential fits and their residuals are shown in black and blue, respectively. Figure S3: Fluorescence trace of hybridisation of 200 nM Cy3-11C to bio15G in air-equilibrated HBS/12 mM DTT. Formation of 8oG after 11 min is followed by a secondary reaction via an intermediate revealed by a long pulse consisting of three discrete fluorescence steps. This suggests the formation of three intermediate species. The final product created after 16 min has no affinity to Cy3-11C, and the pulse pattern terminates. Figure S4: Fluorescence trace of hybridisation of 100 nM Cy3-11G to bio15C in air-equilibrated HBS/12 mM DTT. Oxidation of G in the analyte strand reveals by fluorescence quenching limited to a single pulse. Since no chemical reactions proceed in the immobilised strand, the original pulse pattern continues after dissociation of the oxidised analyte. Figure S5: Fluorescence trace of hybridisation of 300 nM Cy3-12TTT to bio15AAA in air-equilibrated HBS. The dye is stacking to an AT base pair. 8oA (8-oxoA) is a good electron donor and expected to quench Cy3 fluorescence. The trace shows a pulse pattern change similar to that of formation of 8oG. 8oA has a short lifetime and readily reacts to a product that strongly decreases the affinity of the hybrid which is characterised by an ill-defined pattern. From this data we predict that substitution of A by 8oA quenches Cy3 fluorescence by about 30–40%. The affinity of an 8oA modified oligonucleotide should be comparable to that of the parent compound but showing a faster association and dissociation, respectively. The oxidation of A is currently under study. Figure S6: Examples of fluorescence traces demonstrating the remarkable stability of Cy3 in air-equilibrated HBS. (A) Hybridisation of 20 nM 5′Cy3-8 to an Sp modified oligonucleotide (36Sp). (B) Hybridisation of 100 nM 3′Cy3-8 to 36Sp.

## Abbreviations

The following abbreviations are used in this manuscript:

DTT	Dithiothreitol
SPR	Surface Plasmon Resonance
MS	Mass Spectrometry
HPLC	High-Pressure Liquid Chromatography
ZMW	Zero-Mode Waveguide
dsDNA	Double-Strand DNA
ssDNA	Single-Strand DNA
G	Guanosine
8oG	8-Oxo-7,8-dihydroguanosine
Sp	Spiroiminodihydantoin
Gh	5-Guanidinohydantoin
Iz	2,5-Diamino-4H-imidazolone
SMRT	Single-Molecule Real-Time

## References

[1] Simm G.N., Vaucher A.C., Reiher M. (2019). Exploration of Reaction Pathways and Chemical Transformation Networks. J. Phys. Chem. A.

[2] Neeley W.L., Essigmann J.M. (2006). Mechanisms of Formation, Genotoxicity, and Mutation of Guanine Oxidation Products. Chem. Res. Toxicol..

[3] Fleming A.M., Burrows C.J. (2017). Formation and Processing of DNA Damage Substrates for the hNEIL Enzymes. Free Radic. Biol. Med..

[4] Cadet J., Bellon S., Berger M., Bourdat A.-G., Douki T., Duarte V., Frelon S., Gasparutto D., Muller E., Ravanat J.-L. (2002). Recent Aspects of Oxidative DNA Damage: Guanine Lesions, Measurement and Substrate Specificity of DNA Repair Glycosylases. Biol. Chem..

[5] Foote C.S. (1991). Definition of Type I and Type II Photooxidized Oxidation. Photochem. Photobiol..

[6] Steenken S. (1989). Purine-Bases, Nucleosides, and Nucleotides—Aqueous-Solution Redox Chemistry and Transformation Reactions of Their Radical Cations and E- and Oh Adducts. Chem. Rev..

[7] Kobayashi K., Tagawa S. (2003). Direct Observation of Guanine Radical Cation Deprotonation in Duplex DNA Using Pulse Radiolysis. J. Am. Chem. Soc..

[8] Chatgilialoglu C. (2021). The Two Faces of the Guanyl Radical: Molecular Context and Behavior. Molecules.

[9] Burrows C.J., Muller J.G. (1998). Oxidative Nucleobase Modifications Leading to Strand Scission. Chem. Rev..

[10] Shafirovich V., Dourandin A., Huang W., Geacintov N.E. (2001). The Carbonate Radical Is a Site-Selective Oxidizing Agent of Guanine in Double-Stranded Oligonucleotides. J. Biol. Chem..

[11] Ye Y., Muller J.G., Luo W., Mayne C.L., Shallop A.J., Jones R.A., Burrows C.J. (2003). Formation of ^13^C-, ^15^N-, and ^18^O-Labeled Guanidinohydantoin from Guanosine Oxidation with Singlet Oxygen. Implications for Structure and Mechanism. J. Am. Chem. Soc..

[12] Misiaszek R., Crean C., Joffe A., Geacintov N.E., Shafirovich V. (2004). Oxidative DNA Damage Associated with Combination of Guanine and Superoxide Radicals and Repair Mechanisms via Radical Trapping. J. Biol. Chem..

[13] Gervasio F.L., Laio A., Iannuzzi M., Parrinello M. (2004). Influence of DNA Structure on the Reactivity of the Guanine Radical Cation. Chem.-Eur. J..

[14] Shao J., Geacintov N.E., Shafirovich V. (2010). Oxidative Modification of Guanine Bases Initiated by Oxyl Radicals Derived from Photolysis of Azo Compounds. J. Phys. Chem. B.

[15] Ghude P., Schallenberger M.A., Fleming A.M., Muller J.G., Burrows C.J. (2011). Comparison of Transition Metal-Mediated Oxidation Reactions of Guanine in Nucleoside and Single-Stranded Oligodeoxynucleotide Contexts. Inorganica Chim. Acta.

[16] Morikawa M., Kino K., Oyoshi T., Suzuki M., Kobayashi T., Miyazawa H. (2014). Analysis of Guanine Oxidation Products in Double-Stranded DNA and Proposed Guanine Oxidation Pathways in Single-Stranded, Double-Stranded or Quadruplex DNA. Biomolecules.

[17] Rokhlenko Y., Geacintov N.E., Shafirovich V. (2012). Lifetimes and Reaction Pathways of Guanine Radical Cations and Neutral Guanine Radicals in an Oligonucleotide in Aqueous Solutions. J. Am. Chem. Soc..

[18] Rokhlenko Y., Cadet J., Geacintov N.E., Shafirovich V. (2014). Mechanistic Aspects of Hydration of Guanine Radical Cations in DNA. J. Am. Chem. Soc..

[19] Fleming A.M., Xiao S., Chabot M.B., Burrows C.J. (2022). Fluorophore-mediated Photooxidation of the Guanine Heterocycle. J. Phys. Org. Chem..

[20] Fleming A.M., Burrows C.J. (2022). Chemistry of ROS-Mediated Oxidation to the Guanine Base in DNA and Its Biological Consequences. Int. J. Radiat. Biol..

[21] Margolin Y., Shafirovich V., Geacintov N.E., DeMott M.S., Dedon P.C. (2008). DNA Sequence Context as a Determinant of the Quantity and Chemistry of Guanine Oxidation Produced by Hydroxyl Radicals and One-Electron Oxidants. J. Biol. Chem..

[22] Zhu J., Fleming A.M., Orendt A.M., Burrows C.J. (2016). pH-Dependent Equilibrium between 5-Guanidinohydantoin and Iminoallantoin Affects Nucleotide Insertion Opposite the DNA Lesion. J. Org. Chem..

[23] Ravanat J.-L., Saint-Pierre C., Cadet J. (2003). One-Electron Oxidation of the Guanine Moiety of 2′-Deoxyguanosine: Influence of 8-Oxo-7,8-dihydro-2′-deoxyguanosine. J. Am. Chem. Soc..

[24] Lu W., Sun Y., Zhou W., Liu J. (2018). pH-Dependent Singlet O_2_ Oxidation Kinetics of Guanine and 9-Methylguanine: An Online Mass Spectrometry and Spectroscopy Study Combined with Theoretical Exploration. J. Phys. Chem. B.

[25] Osakada Y., Kawai K., Tachikawa T., Fujitsuka M., Tainaka K., Tero-Kubota S., Majima T. (2012). Generation of Singlet Oxygen during Photosensitized One-Electron Oxidation of DNA. Chem.-Eur. J..

[26] Kasai H., Yamaizumi Z., Berger M., Cadet J. (1992). Photosensitized Formation of 7,8-Dihydro-8-Oxo-2′-Deoxyguanosine (8-Hydroxy-2′-Deoxyguanosine) in DNA by Riboflavin: A Nonsinglet Oxygen-Mediated Reaction. J. Am. Chem. Soc..

[27] Ghosh A.K., Schuster G.B. (2006). Role of the Guanine N1 Imino Proton in the Migration and Reaction of Radical Cations in DNA Oligomers. J. Am. Chem. Soc..

[28] Yun B.H., Lee Y.A., Kim S.K., Kuzmin V., Kolbanovskiy A., Dedon P.C., Geacintov N.E., Shafirovich V. (2007). Photosensitized Oxidative DNA Damage: From Hole Injection to Chemical Product Formation and Strand Cleavage. J. Am. Chem. Soc..

[29] Sheu C., Kang P., Khan S., Foote C.S. (2002). Low-Temperature Photosensitized Oxidation of a Guanosine Derivative and Formation of an Imidazole Ring-Opened Product. J. Am. Chem. Soc..

[30] Kang P., Foote C.S. (2002). Formation of Transient Intermediates in Low-Temperature Photosensitized Oxidation of an 8-^13^C-Guanosine Derivative. J. Am. Chem. Soc..

[31] Candeias L.P., Steenken S. (1989). Structure and Acid-Base Properties of One-Electron-Oxidized Deoxyguanosine, Guanosine, and 1-Methylguanosine. J. Am. Chem. Soc..

[32] Steenken S., Jovanovic S. (1997). How Easily Oxidizable Is DNA? One-Electron Reduction Potentials of Adenosine and Guanosine Radicals in Aqueous Solution. J. Am. Chem. Soc..

[33] Sobek J., Rehrauer H., Schauer S., Fischer D., Patrignani A., Landgraf S., Korlach J., Schlapbach R. (2016). Single-Molecule DNA Hybridisation Studied by Using a Modified DNA Sequencer: A Comparison with Surface Plasmon Resonance Data. Methods Appl. Fluoresc..

[34] Sobek J., Schmidt M., Grossmann J., Rehrauer H., Schmidt L., Schlapbach R. (2020). Single-Molecule Chemistry. Part I: Monitoring Oxidation of G in Oligonucleotides Using CY3 Fluorescence. Methods Appl. Fluoresc..

[35] Eid J., Fehr A., Gray J., Luong K., Lyle J., Otto G., Peluso P., Rank D., Baybayan P., Bettman B. (2009). Real-Time DNA Sequencing from Single Polymerase Molecules. Nat. Methods.

[36] Korlach J., Bjornson K.P., Chaudhuri B.P., Cicero R.L., Flusberg B.A., Gray J.J., Holden D., Saxena R., Wegener J., Turner S.W. (2010). Chapter 20—Real-Time DNA Sequencing from Single Polymerase Molecules.

[37] Gidi Y., Ramos-Sanchez J., Lovell T.C., Glembockyte V., Cheah I.K., Schnermann M.J., Halliwell B., Cosa G. (2023). Superior Photoprotection of Cyanine Dyes with Thio-Imidazole Amino Acids. J. Am. Chem. Soc..

[38] Schwechheimer C., Rönicke F., Schepers U., Wagenknecht H.-A. (2018). A New Structure–Activity Relationship for Cyanine Dyes to Improve Photostability and Fluorescence Properties for Live Cell Imaging. Chem. Sci..

[39] Cisse I.I., Kim H., Ha T. (2012). A Rule of Seven in Watson-Crick Base-Pairing of Mismatched Sequences. Nat. Struct. Mol. Biol..

[40] Sobek J., Schlapbach R. (2020). Dependence of Fluorescence Quenching of CY3 Oligonucleotide Conjugates on the Oxidation Potential of the Stacking Base Pair. Molecules.

[41] Mujumdar R.B., Ernst L.A., Mujumdar S.R., Lewis C.J., Waggoner A.S. (1993). Cyanine Dye Labeling Reagents: Sulfoindocyanine Succinimidyl Esters. Bioconjug. Chem..

[42] Sanborn M.E., Connolly B.K., Gurunathan K., Levitus M. (2007). Fluorescence Properties and Photophysics of the Sulfoindocyanine Cy3 Linked Covalently to DNA. J. Phys. Chem. B.

[43] Harvey B.J., Levitus M. (2009). Nucleobase-Specific Enhancement of Cy3 Fluorescence. J. Fluoresc..

[44] Harvey B.J., Perez C., Levitus M. (2009). DNA Sequence-Dependent Enhancement of Cy3 Fluorescence. Photochem. Photobiol. Sci..

[45] Henary M., Mojzych M. (2008). Stability and Reactivity of Polymethine Dyes in Solution. Topics in Heterocyclic Chemistry.

[46] Zheng Q., Juette M.F., Jockusch S., Wasserman M.R., Zhou Z., Altman R.B., Blanchard S.C. (2014). Ultra-Stable Organic Fluorophores for Single-Molecule Research. Chem. Soc. Rev..

[47] Chen C., Zhou B., Lu D., Xu G. (1995). Electron Transfer Events in Solutions of Cyanine Dyes. J. Photochem. Photobiol. Chem..

[48] Demchenko A.P. (2020). Photobleaching of Organic Fluorophores: Quantitative Characterization, Mechanisms, Protection. Methods Appl. Fluoresc..

[49] Spiriti J., Binder J.K., Levitus M., Van Der Vaart A. (2011). Cy3-DNA Stacking Interactions Strongly Depend on the Identity of the Terminal Basepair. Biophys. J..

[50] Niles J.C., Wishnok J.S., Tannenbaum S.R. (2001). Spiroiminodihydantoin Is the Major Product of the 8-Oxo-7,8-Dihydroguanosine Reaction with Peroxynitrite in the Presence of Thiols and Guanosine Photooxidation by Methylene Blue. Org. Lett..

[51] Ravanat J.-L., Martinez G.R., Medeiros M.H.G., Di Mascio P., Cadet J. (2006). Singlet Oxygen Oxidation of 2′-Deoxyguanosine. Formation and Mechanistic Insights. Tetrahedron.

[52] Gao Y., Wolf L.K., Georgiadis R.M. (2006). Secondary Structure Effects on DNA Hybridization Kinetics: A Solution versus Surface Comparison. Nucleic Acids Res..

[53] Macedo L.J.A., Miller E.N., Opdahl A. (2017). Effect of Probe-Probe Distance on the Stability of DNA Hybrids on Surfaces. Anal. Chem..

[54] Treasurer E., Levicky R. (2021). How Surfaces Affect Hybridization Kinetics. J. Phys. Chem. B.

[55] Levene M.J., Korlach J., Turner S.W., Foquet M., Craighead H.G., Webb W.W. (2003). Zero-Mode Waveguides for Single-Molecule Analysis at High Concentrations. Science.

[56] Steenken S., Jovanovic S.V., Bietti M., Bernhard K. (2000). The Trap Depth (in DNA) of 8-Oxo-7,8-Dihydro-2′deoxyguanosine as Derived from Electron-Transfer Equilibria in Aqueous Solution. J. Am. Chem. Soc..

[57] Adam W., Saha-Möller C.R., Schonberger A. (1996). Photooxidation of 8-Oxo-7,8-Dihydro-2′-Deoxyguanosine by Thermally Generated Triplet-Excited Ketones from 3-(Hydroxymethyl)-3,4,4-Trimethyl-1,2-Dioxetane and Comparison with Type I and Type II Photosensitizers. J. Am. Chem. Soc..

[58] Sheu C., Foote C.S. (1995). Reactivity toward Singlet Oxygen of a 7,8-Dihydro-8-Oxoguanosine (“8-Hydroxyguanosine”) Formed by Photooxidation of a Guanosine Derivative. J. Am. Chem. Soc..

[59] Schweitzer C., Schmidt R. (2003). Physical Mechanisms of Generation and Deactivation of Singlet Oxygen. Chem. Rev..

[60] Ravanat J., Dumont E. (2022). Reactivity of Singlet Oxygen with DNA, an Update. Photochem. Photobiol..

[61] Devasagayam T.P.A., Sundquist A.R., Di Mao P., Kaiser S., Sies H. (1991). Activity of Thiols as Singlet Molecular-Oxygen Quenchers. J. Photochem. Photobiol. B.

[62] Gasper S.M., Schuster G.B. (1997). Intramolecular Photoinduced Electron Transfer to Anthraquinones Linked to Duplex DNA: The Effect of Gaps and Traps on Long-Range Radical Cation Migration. J. Am. Chem. Soc..

[63] Misiaszek R., Uvaydov Y., Crean C., Geacintov N.E., Shafirovich V. (2005). Combination Reactions of Superoxide with 8-Oxo-7,8-Dihydroguanine Radicals in DNA: Kinetics and end products. J. Biol. Chem..

[64] Ikeda H., Saito I. (1999). 8-Methoxydeoxyguanosine as an Effective Precursor of 2-Aminoimidazolone, a Major Guanine Oxidation Product in One-Electron Oxidation of DNA. J. Am. Chem. Soc..

[65] Fleming A.M., Armentrout E.I., Zhu J., Muller J.G., Burrows C.J. (2015). Spirodi(Iminohydantoin) Products from Oxidation of 2′-Deoxyguanosine in the Presence of NH_4_Cl in Nucleoside and Oligodeoxynucleotide Contexts. J. Org. Chem..

[66] McCallum J.E.B., Kuniyoshi C.Y., Foote C.S. (2004). Characterization of 5-Hydroxy-8-Oxo-7,8-Dihydroguanosine in the Photosensitized Oxidation of 8-Oxo-7,8-Dihydroguanosine and Its Rearrangement to Spiroiminodihydantoin. J. Am. Chem. Soc..

