# Supplementary figures and images for: Single-Molecule Chemistry Part II: Pathway Analysis of the Oxidation of Guanine to 8-Oxo-7,8-dihydroguanosine in an Oligonucleotide Hybrid
Source: Molecules. 2026 May 8;31(10):1564. doi: 10.3390/molecules31101564 (PMC13210223; doi:10.3390/molecules31101564)

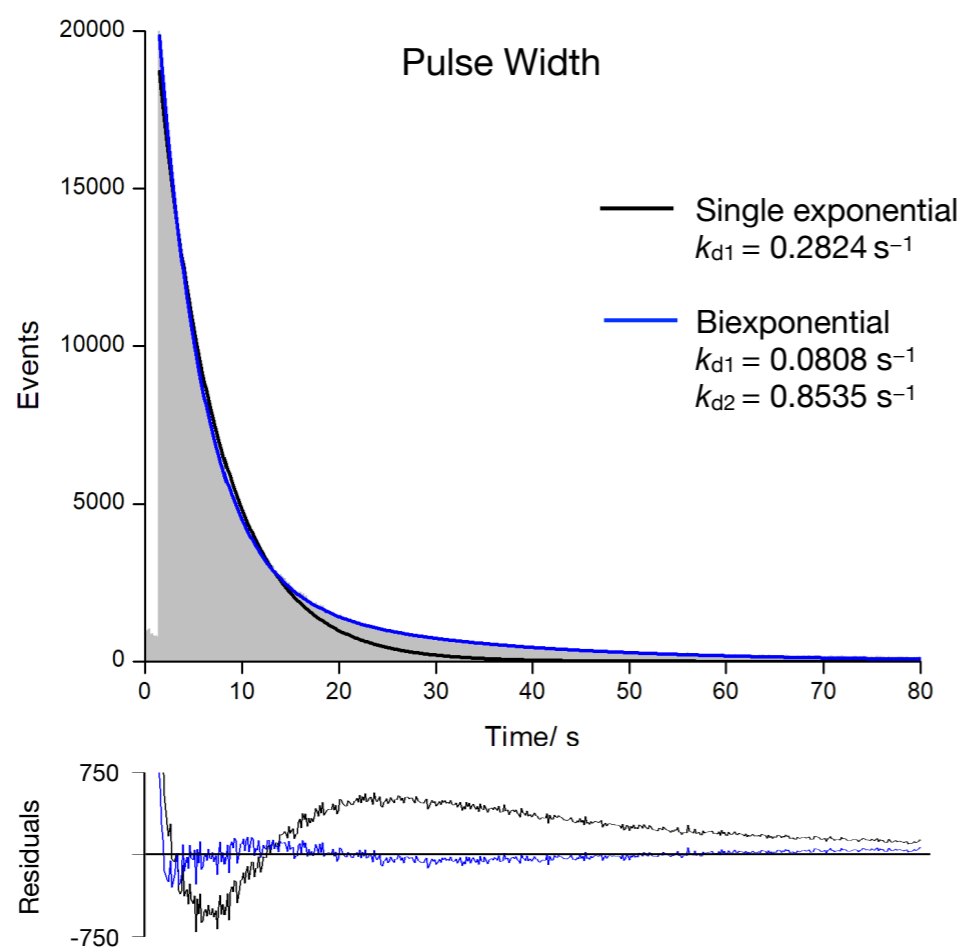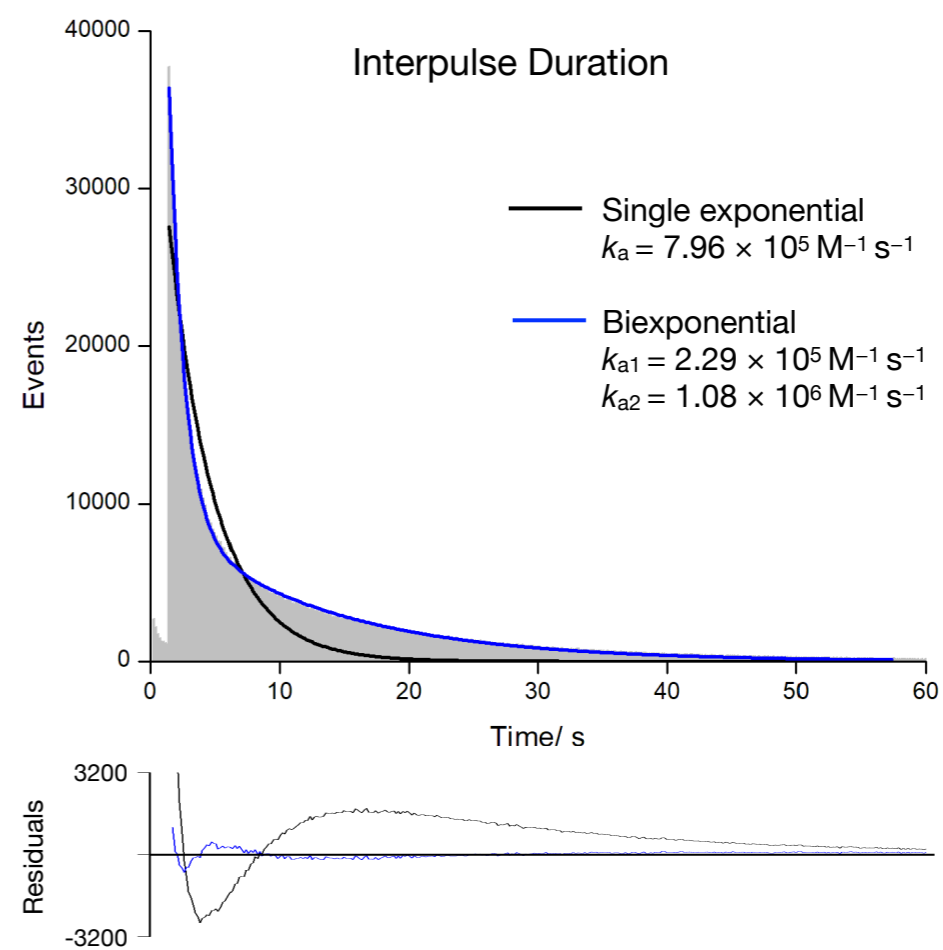

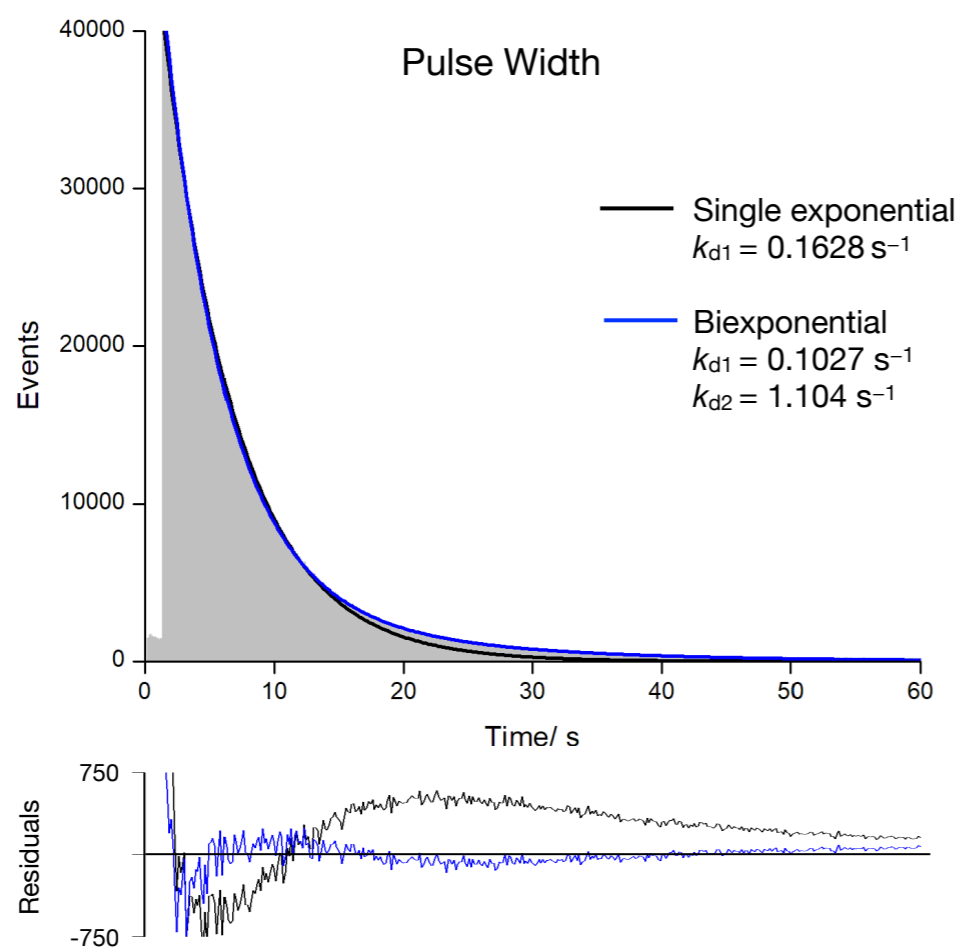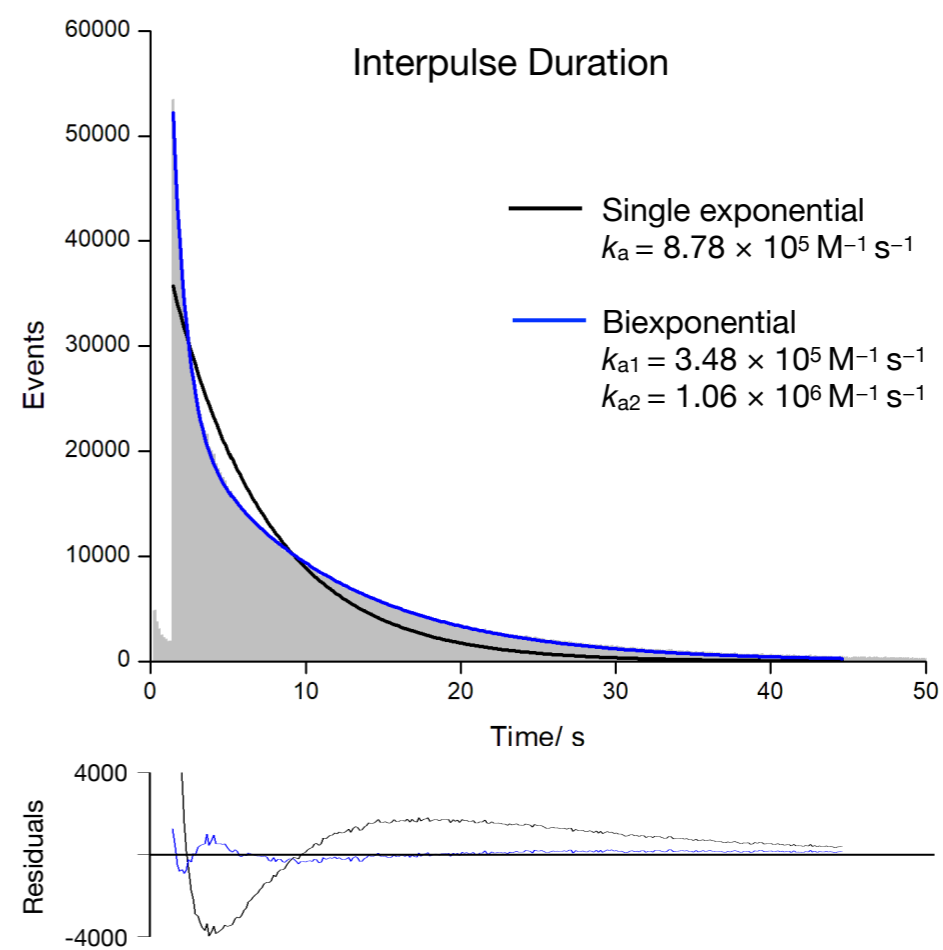

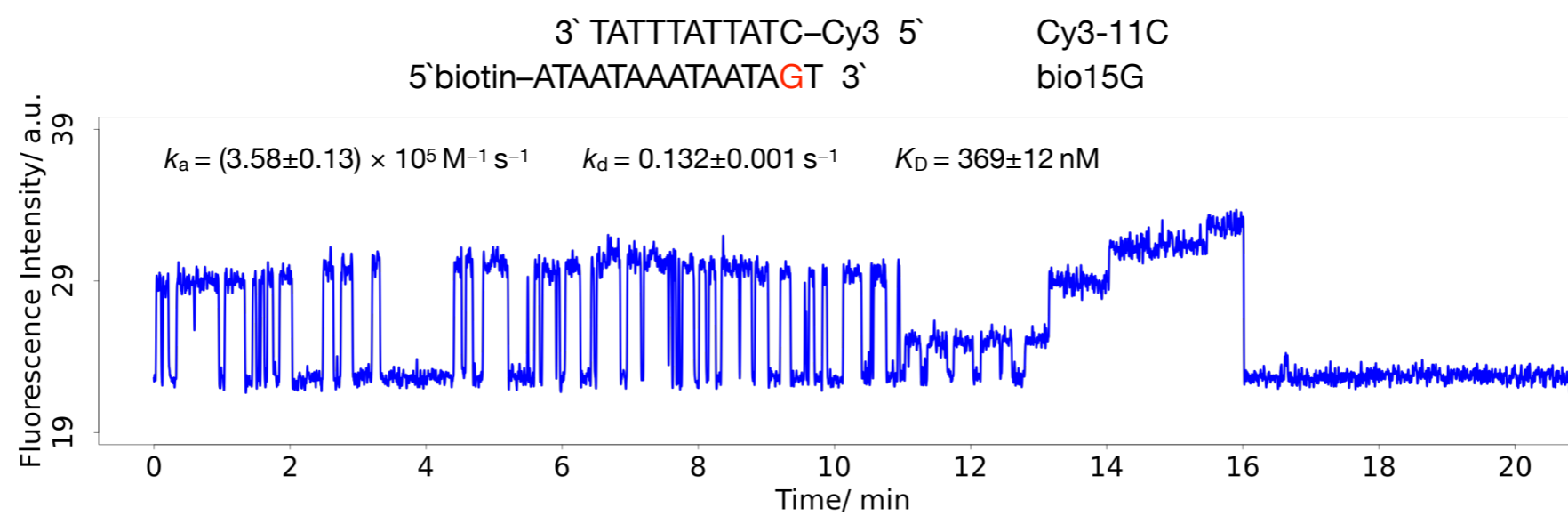

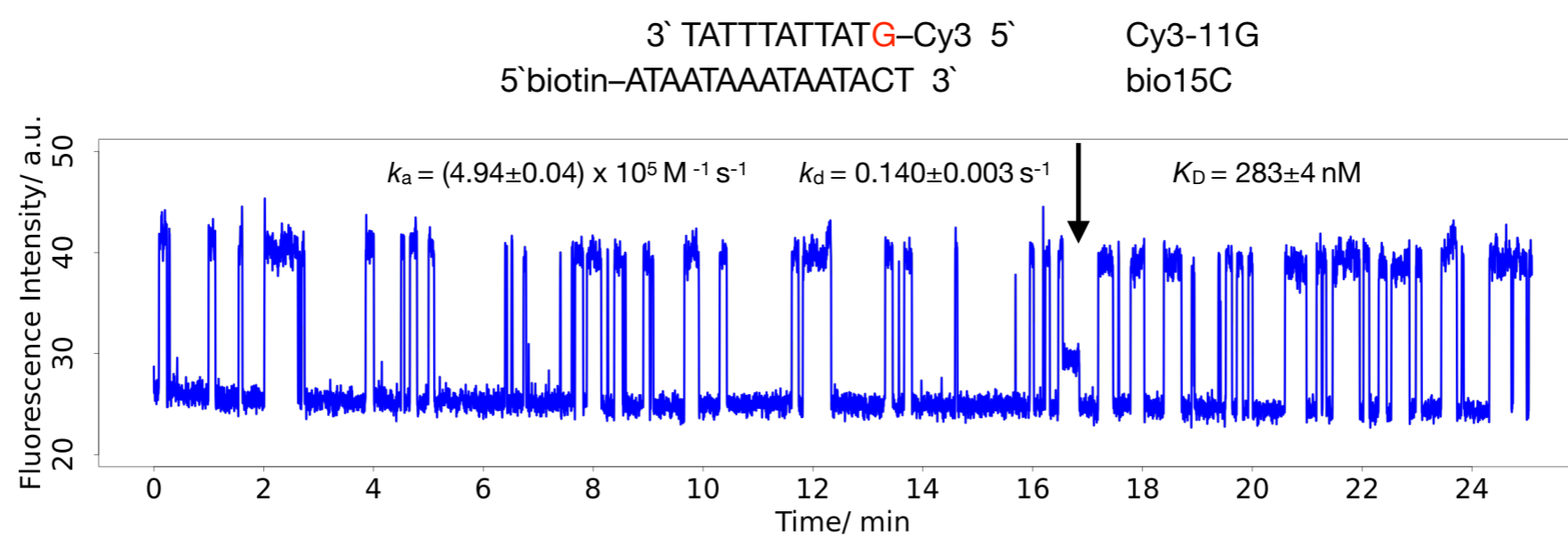

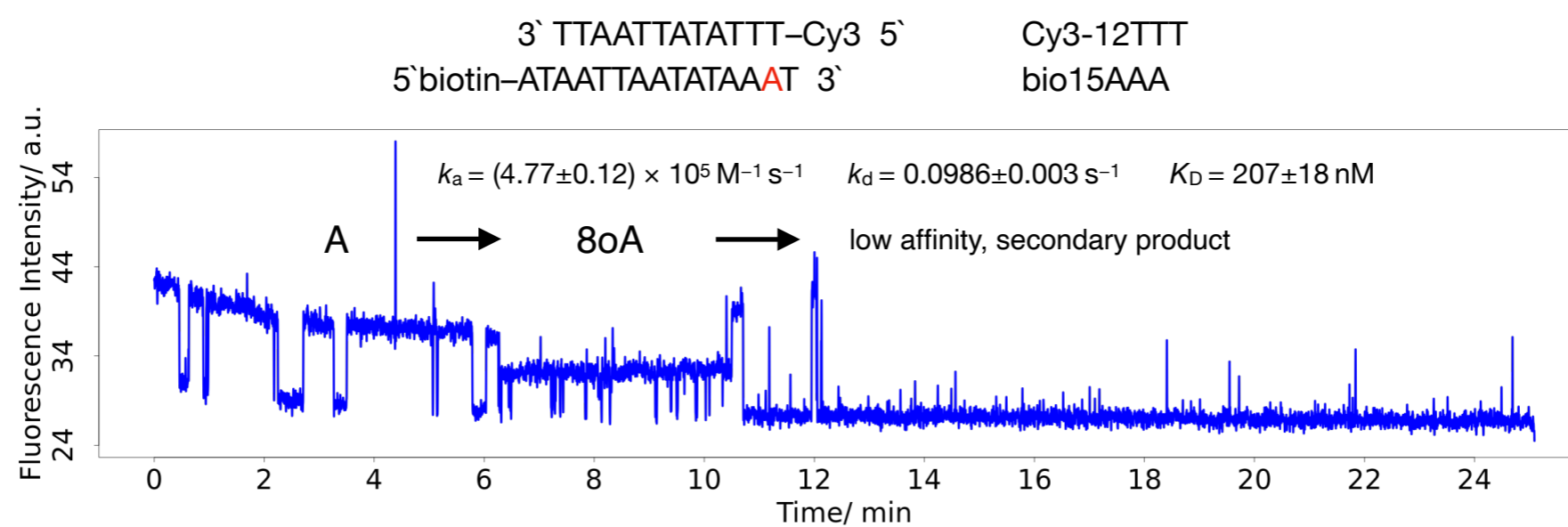

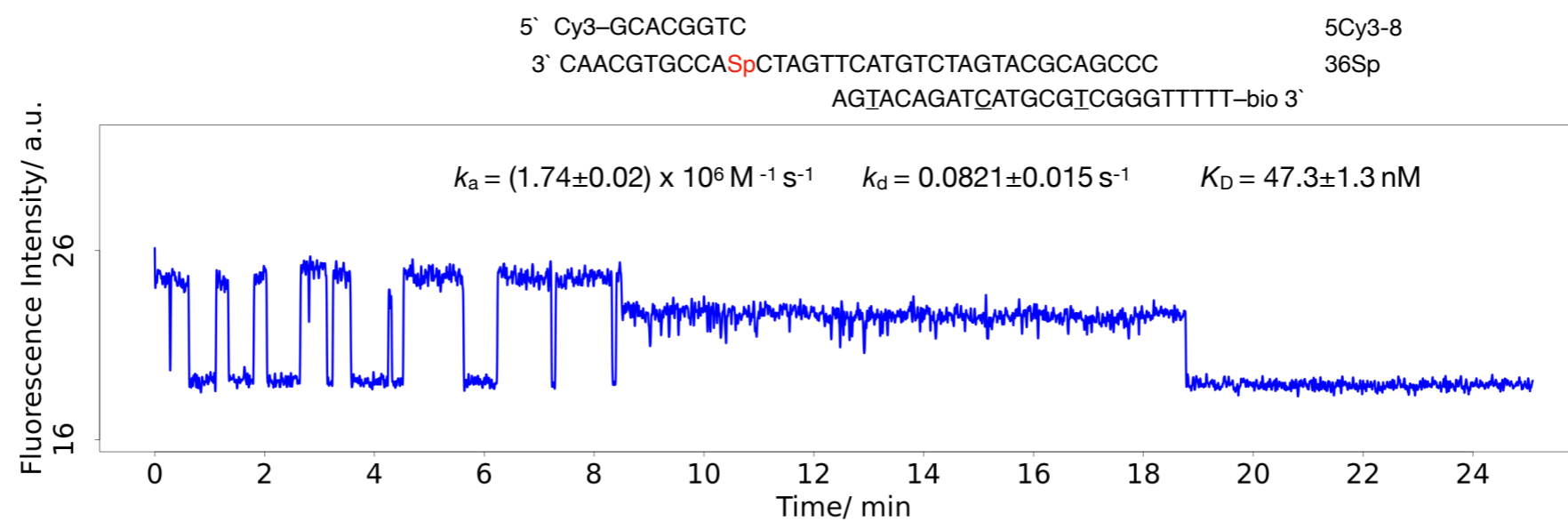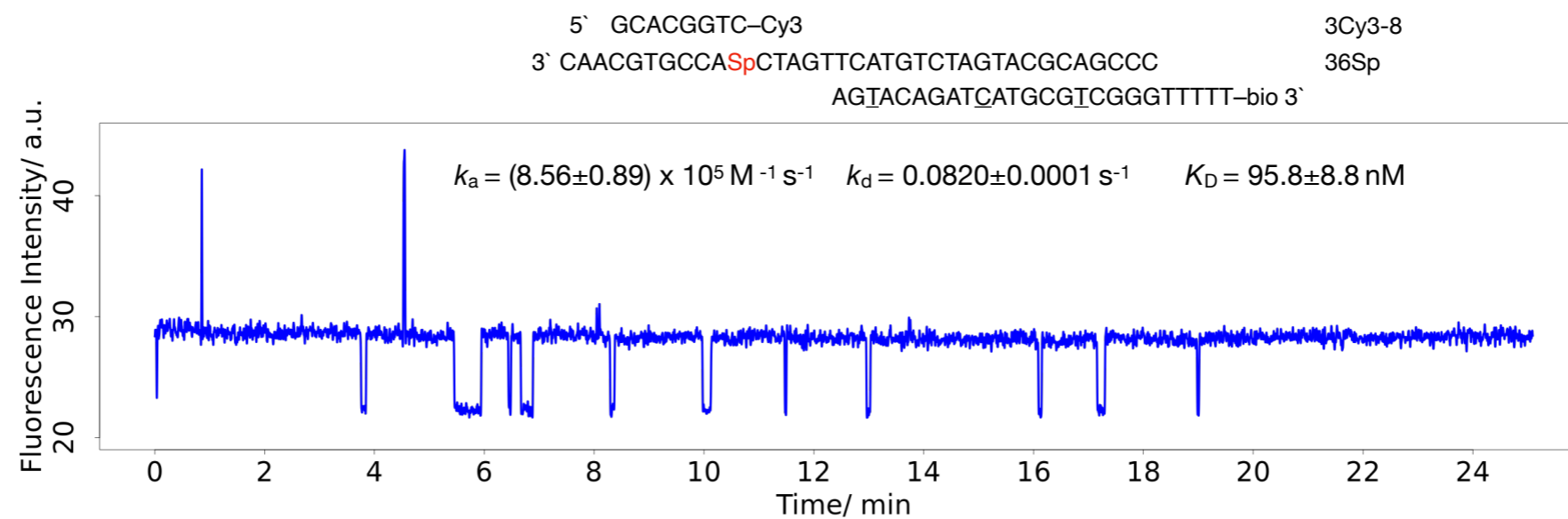

Supplement: Supplementary file 1 [file molecules-31-01564-s001.zip › molecules-4253661-supplementary.pdf]
